# Comparation of EGFR-TKI (EGFR tyrosine kinase inhibitors) combination therapy and osimertinib for untreated EGFR-mutated advanced non-small cell lung cancers: A systematic review and network meta-analysis

**DOI:** 10.1097/MD.0000000000034483

**Published:** 2023-07-28

**Authors:** Yang Lei, Jia Duan, Qiong Zhang, Qing Li

**Affiliations:** a Department of Geriatrics, Neijiang First People’s Hospital, Neijiang, China; b Department of Intensive Care Medicine, Neijiang Hospital of Traditional Chinese Medicine, Neijiang, China.

**Keywords:** anti-angiogenic, combination therapy, non-small cell lung cancers, osimertinib

## Abstract

EGFR-TKI (tyrosine kinase inhibitor) monotherapy has become the first-line treatment option for patients with EGFR-mutated non-small cell lung cancer (NSCLC). Prolonging the survival time, improving the progression-free survival of front-line treatment, and delaying the occurrence of drug resistance. At present, combination therapy is being widely used. Evaluate the therapeutic effect of TKI joint and Osimertinib drug therapy for positive patients with gene positive.

**Material and methods:**

Articles that met the inclusion criteria were searched through electronic databases. treatment emergent adverse events were summarized, and progression-free survival (PFS) and overall survival (OS) were calculated. Appropriate networks for different outcomes were created to incorporate all the evidence. Bayesian network-based multitreatment was used to compare the efficacy and specific toxicity of all treatment regimens.

**Results:**

Fourteen eligible studies involving 2325 patients were included. Of these, 7 studies compared EGFR-TKI plus chemotherapy with EGFR-TKI alone, and 6 studies compared EGFR-TKI plus antiangiogenic therapy with EGFR-TKI alone. One study compared Osimertinib and GP, ER, EB, and GCP were more effective than SOC in PFS analysis; however, there was no significant difference between osimertinib and the other 4 combination regimens. The cumulative probabilities of being the most efficacious treatments were (PFS, OS, treatment emergent adverse events): O (73%, 16%, 0%, 0%), GCP (14%, 64%, 10%, 16%), GP (2%, 17%,8%), and EB (3%, 3%, 8%), ER (5%, NA, 4%);GA(1%, NA, 69%).

**Conclusion:**

Osimertinib has the lowest side effects and provides better PFS first-line treatment in advanced EGFR-mutated NSCLC.GCP is the best regimen for OS, but its toxicity limits its application, and it may be the first choice for patients with higher survival requirements.

## 1. Introduction

Lung cancer is one of the tumors with the highest morbidity and mortality, and non-small cell lung cancer (NSCLC) accounts for about 85% of overall reported cases.^[1]^ EGFR is a member of the ErbB receptor tyrosine kinase (TK) family, which plays a crucial role in the progression of NSCLC.^[2]^ EGFR signaling has been reported to affect angiogenesis, activation and regulation of cell proliferation, and epithelial-mesenchymal transition. At present, it is believed that the EGFR mutation rate can reach up to 50% in nonsmokers, adenocarcinoma patients, Asian patients, and female patients.^[3]^ The most common EGFR mutations were exon 19 deletions and L858r point mutations in exon 21.^[4]^ Since the IPASS study in 2003.^[5]^ EGFR-TKI monotherapy has become the first-line treatment option for EGFR-mutated non-small cell lung cancer, and multiple generation EGFR-TKIs have been developed (including erlotinib, gefitinib, icotinib (first generation),dacomitinib and afatinib (second generation), and osimertinib (third generation). A series of RCTs have confirmed the non-inferior efficacy and relatively low toxicity of erlotinib.^[6]^ icotinib^[7,8]^ and gefitinib^[5]^ in the treatment of NSCLC patients compared with standard chemotherapy and most of these TKIs have been established as standard first line treatments.^[9]^ However, durable responses to EGFR-TKIs remain a perennial challenge for the inevitable development of acquired resistance, and biologically synergistic combinations of EGFR-TKIs with other treatments with different mechanisms of action, including chemotherapy and monoclonal antibodies, are first-line options to overcome resistance and prolong survival.^[10]^ Currently, the mainstream treatment is TKI combined with double-drug chemotherapy or anti-angiogenic drugs. The combination therapy with first-generation TKI is due to its tolerable side effects, the median survival without infusion of osimertinib, and the possibility of subsequent use of osimertinib. The JO25567 trial (JapicCT-111390) identified that the addition of bevacizumab to erlotinib demonstrated a significant clinical benefit in improving PFS(16.0 vs 9.7 months).^[11]^ Similarly, the NEJ009 study (UMIN000006340) shows that concurrent combined treatment of gefitinib and chemotherapy significantly extends both PFS (20.9 vs 11.9 months) and overall survival (OS) (50.9 vs 38.8months) compared with EGFR-TKI monotherapy.^[12]^ The National Comprehensive Cancer Network and European Society for Medical Oncology (ESMO) guidelines recommend first-line osimertinib as the preferred option and other treatment strategies as alternative candidates.^[9,13]^Combination therapy with a first-generation TKI is a promising treatment option, and a subgroup of patients who may benefit from this therapy was screened.

## 2. Methods

### 2.1. Registration

This meta-analysis was registered with PROSPERO under the registration ID:CRD42022358706. Detailed Project website: https://www.crd.york.ac.uk/prospero/display_record.php?ID=CRD42022358706.

### 2.2. Retrieval strategy

Three electronic databases (PubMed, EMBASE, and the Cochrane Library) were searched from inception to August 31, 2022, using Medical Subject Headings (MeSH) and text words. The language was restricted to English, and there were no restrictions on whether or not to publish; however, complete data were required. Additional studies were sought in the reference lists of all identified publications, including relevant meta-analyses and systematic reviews, and the detailed search strategy was presented in PROSPERO.

### 2.3. Inclusion criteria

The included RCTS included patients with confirmed stage III/IV NSCLC carrying EGFR mutations [mainly exon 19 deletion (19del) and exon 21 L858R mutation (L858R). All RCTS compared EGFR-TKI based combination therapy or osimertinib versus EGFR-TKI monotherapy as the first-line therapy.

### 2.4. Outcomes

Primary endpoint are progression free survival (PFS). Secondary end points are (treatment emergent adverse events) TEAEs and OS.

### 2.5. Data extraction

Two authors (J.D. and L.Y.) reviewed titles and abstracts independently. If a study met all the inclusion criteria, the full text retrieved was used for subsequent evaluation, the details of the eligible studies were tabulated by 2 authors (J.D. and L.Y.), and the extracted data were confirmed and evaluated by 2 other authors (L.Y. and Q.Z.). We prioritized the extraction of survival data assessed by an independent review committee to avoid potential assessment bias. Hazard ratios (HRS) and their 95% confidence intervals (CIs) for PFS and OS were extracted for adverse events (TEAEs) occurring during treatment of grade 3 or worse.

If an RCT published more than one article for analysis, we extracted all data and summarized the latest results for data analysis. The reliability of the extracted results was verified by using the corresponding trial registry of each study. Disagreements were resolved by re-reading the original text and discussion and contacting the original authors, if necessary, until a consensus was reached.

### 2.6. Quality assessment

Two authors (L.Y. and Q.Z.) assessed risk of bias was assessed for each study using an assessment tool.^[14]^ Randomization sequence generation, concealment of assignments, blinding of participants and personnel, blinding of outcome assessments, incomplete outcome data, selective reporting, and other biases were also evaluated. Differences were resolved through consultation with other members. RevMan 5.3 software provided by Cochrane Collaboration, was used to draw the “risk of bias map” and “risk of bias summary.”

### 2.7. Statistical analysis

Meta-analysis was performed using R 4.2.2 and Rstudio software. The chi-square test and *I*^2^ test were used to evaluate the heterogeneity among the included studies. There was no statistical heterogeneity among the studies (*P* > .05, *I*^2^ ≤ 50%), and a fixed effects model was used. If there was heterogeneity, we performed subgroup and sensitivity analyses to determine which studies contributed to heterogeneity, followed by elimination of the remaining studies and recalculation of the pooled estimates. TEAEs were analyzed using the risk ratio (RR). PFS and OS were combined and analyzed using hazard ratio (HR). The corresponding 95% confidence interval (95%CI) was calculated, and statistical significance was set at *P* < .05. Using JAGS and R GeMTC package of GeMTC (https://drugis.org/software/r-packages/), the random effects model in (the generalized linear model [GLM]) Bayesian network meta-analysis of all ending on (NMA). For each outcome measure, 4 independent Markov chains were simultaneously used for 5000 burn-ins and 20,000 inference iterations per chain to obtain the posterior distribution. The trace plot and Brooks-Gelman-Rubin methods were used to assess the convergence of the model. The treatment effects were estimated using HR/OR and the corresponding 95% CI. Network consistency was assessed using node-split models by statistically testing direct and indirect estimates within the treatment loop. To rank the probabilities of all available treatments, the area under the cumulative ranking curve (SUCRAs) was calculated and logarithmic. SUCRA greater than zero indicates that the treatment is better, and if it is less than zero, it is worse. To compare the efficacy and tolerability of the combination regimen and osimertinib versus SOC and to assess their benefit-risk ratios, we also ranked them according to the SUCRA values of PFS, OS, and tolerability (1-SUCRASAEs) in the ranking AND Rank Probabilities.

## 3. Results

### 3.1. Study selection

**Figure 1.**
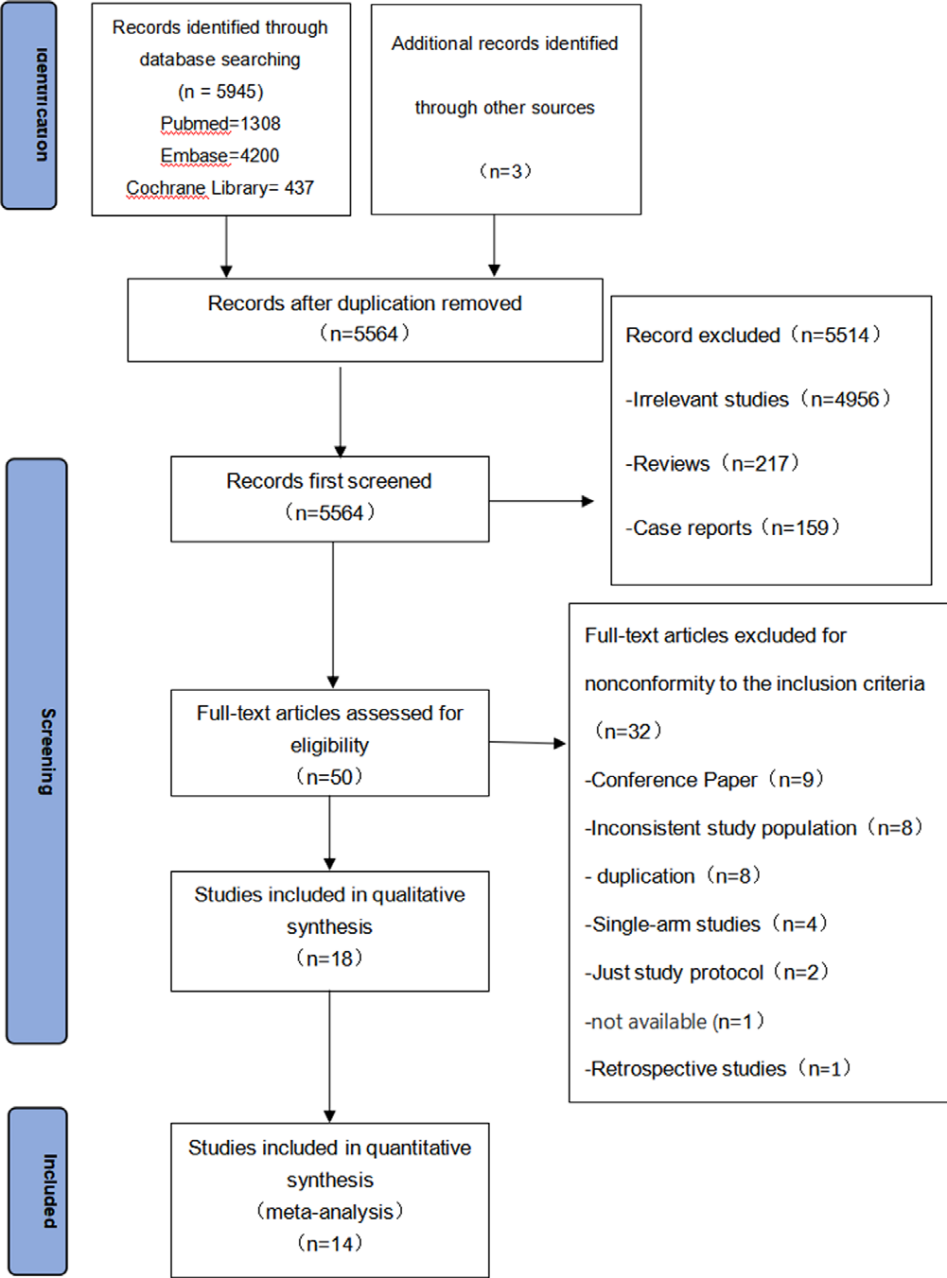
Literature search and selection.

Figure 1 presents the literature screening flowchart. After an initial database search (Pubmed 1308, Embase 4200, Cochrane Library 437, and other sources (meta-analysis) 3), 5945 articles were included. After duplicates were removed, 5564 articles remained. We then read the titles and abstracts and further excluded 5514 articles, 4956 is irrelevant studies, 217 reviews, 159 case reports, 59 retrospective studies, and 114 Meta-analyses; 50 of full-text articles assessed for eligibility, After reading the full text and discussions among members, 32 articles were excluded, including 9 conference papers, 8 studies involving inconsistent study population, 8 duplicate articles, 4 single-arm studies, 2 study protocol,1 not available, and 1 studies retrospective studies, At last, 14 RCTs (22 references)^[10–12,16–33]^were included.

### 3.2. Quality assessment

The risk of bias among the included studies was assessed using the Cochrane Intervention System Review Manual, including random sequence generation, allocation concealment, double blinding of researchers and participants, blinding of outcome assessment, incomplete outcome data, and selective reporting; each study was qualified as high, low, or unclear risk of bias.

### 3.3. Characteristics of included studies

**Figure 2.**
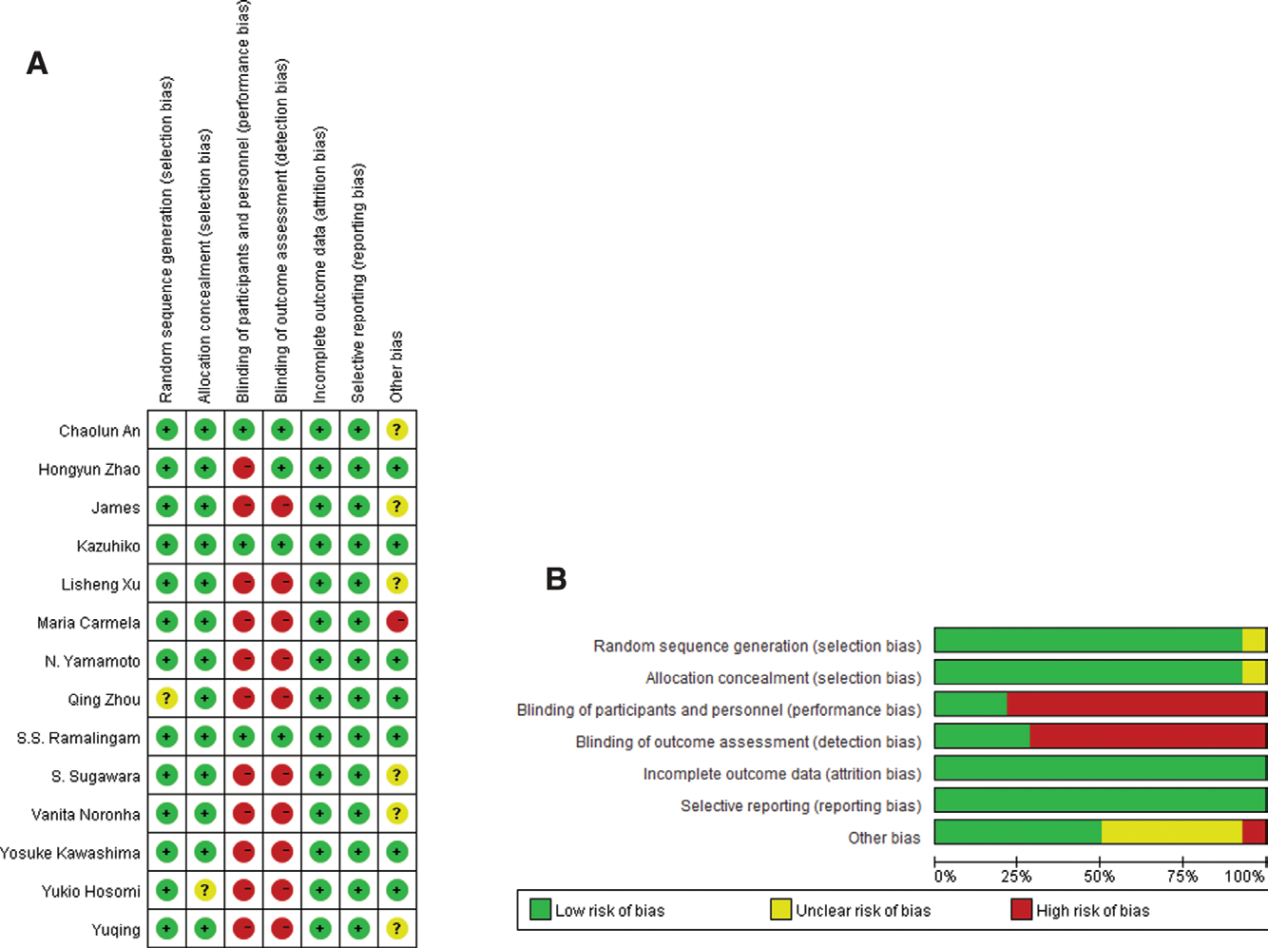
A. Risk of bias summary. B. Risk of bias graph.

Finally, 14 eligible studies involving 2325 patients were included. Of these, 7 studies compared EGFR-TKI plus chemotherapy with EGFR-TKI alone, and 6 studies compared EGFR-TKI plus antiangiogenic therapy with EGFR-TKI alone. One study compared Osimertinib with EGFR-TKI alone. Specifically, the study “Yuqing Lou” involved 3 groups. The patients received first-generation SOC plus pemetrexed and carboplatin, pemetrexed plus carboplatin, or gefitinib monotherapy. Given our meta-analysis inclusion criteria, we present only the results for the first 2 groups. In the overall population, 1109 patients (47.7) received combination therapy, 279 (12%) received osimertinib, and 1368 (58.8%) were female. The characteristics of these studies are summarized in Table 1 and Table S1, Supplemental Digital Content, http://links.lww.com/MD/J360, which is an additional complement to the original trial data, including ORR, PFS, OS, TEAEs. Figure 2A and B shows the results of the quality assessment of the included studies.

**Table 1:** Clinical characteristics of included trials.

Study	Sample size	Phase	Center	Region	Treatment	Year	Gender		Age	EGFR mutation		PFS	OS	
							male	Female		19 del	21L858R			
Chaolun An	45	II	Multicenter	China	GP	2010–2013	25	20	65.72 ± 13.02	17	28	18.0 (95% CI, 15.7–16.2)	34 (95% CI, 28.7.7–39.2)	
	45				SOC		25	20	66.89 ± 12.46	16	29	14.0 (95% CI, 11.8–16.2)	32 (95% CI, 26.7–37.2)	
Yukio Hosomi,	169	III	Multicenter	Global	GCP	2011–2015	56	114	64.8 ± 7.8	93	69	20.93 (95% CI, 17.94–24.20)	50.90 (95% CI, 41.77–62.50)	
	172				SOC		64	108	64.0 ± 8.4 (37–75)	95	67	11.17 (95% CI, 8.97–13.40)	38.80 (95% CI, 31.10–47.33)	
S.S. Ramalingam	279	III	Multicenter	Global	O	2014–2016	101	178	64.0 (26.0–85.0)	175	104	18.9 (95% CI, 15.2–21.4)	38.6 (95% CI, 34.5–41.8)	
	277				SOC		105	172	64.0 (35.0–93.0)	174	103	10.2 (95% CI, 9.6–11.1)	31.8 (95% CI, 26.6–36.0)	
James Chih-Hsin Yang,	129	II	Multicenter	East Asian	GP	2012–2013	64	82	62.0 (33.2–83.6)	65	52	16.2 (95% CI, 12.6–18.7)	43.4 (95% CI, 33.4–50.8)	
	66				SOC		24	41	62.0 (40.5–79.5)	40	23	11.1 (95% CI, 9.7–13.8)	36.8 (95% CI, 26.7–42.6).	
Kazuhiko Nakagawa	224	III	Multicenter	Global	ER	2016–2018	83	141	65.0 (57.0–71.0)	123	99	19.4 (95% CI, 15.4–21.6)	NR	
	225				SOC		83	142	64.0 (56.0–70.0)	120	105	12.4 (95% CI, 11.0–13.5)	NR	
N. Yamamoto	75	II	Multicenter	Japan	EB	2011–2012	30	45	67.0 (59.0–73.0)	40	35	16.0 (95% CI, 13.9–18.1)	47	
	77				SOC		26	51	67.0 (60.0–73.0)	40	37	9.7 (95% CI, 5.7–11.1)	47.4	
Hongyun Zhao	157	III	Multicenter	China	GA	2017–2018	66	91	57.0 (51.0–65.0)	81	74	13.7 (95% CI, 11.9–14.1)	NR	
	156				SOC		62	94	60.0 (51.0–65.0)	83	73	10.2 (95% CI, 10.1–11.9)	NR	
Qing Zhou	157	III	Multicenter	China	EB	2016–2017	41	71	67.0 (61.0–73.0)	82	75	17.9 (95% CI, 15.2–19.9)	36.2 (95% CI, 32.5–42.4)	
	154				SOC		39	73	68.0 (62.0–73.0)	79	75	11.2 (95% CI, 9.7–13.8)	31.6 (95% CI, 27.2–40.0)	
Yosuke Kawashima	112	III	Multicenter	Japan	EB	2015– 2016	41	71	67.0 (61.0–73.0)	56	56	16.9 (95% CI;14.2–21.0)	50.7 (95% CI, 37.3–NE)	
	114				SOC		39	73	68.0 (62.0–73.0)	55	57	13.3 (95% CI, 11.1–15.3)	46.2 (95% CI, 38.2–NE)	
Maria Carmela Piccirillo	80	III	Multicenter	Italy	EB	2016– 2019	28	52	65.9 (57.9–71.8)	44	32	15.4 (95% CI, 12.2–18.6)	33.3 (95% CI, 24.3–45.1)	
	80				SOC		30	50	67.7 (60.7–73.6)	44	34	9.6 (95% CI, 8.2–10.6)	22.8 (95% CI, 18.3–33.0)	
Yuqing Lou,Baohui Han	40	II	Monocenter	China	GCP	2011–2015	15	25	< 65.0 (57.5%)	21	19	17.5 (95% CI, 15.3–19.7)	37.9 (95% CI, 17.3–58.6)	
	41				SOC		18	23	< 65.0 (43.9%)	21	20	11.9 (95% CI, 9.1–14.6)	25.8 (95% CI,19.2–32.3)	
Vanita Noronha,	174	III	Monocenter	India	GCP	2016–2018	88	96	54.0 (27.0–75.0)	107	109	16.0 (95% CI, 13.5–18.5)	NR	
	176				SOC		93	83	56.0 (27.0–78.0)	60	60	8.0 (95% CI, 7.0–9.0)	17 (95% CI, 13.5–20.5)	
S. Sugawara	41	II	Multicenter	Japan	GCP	2010–2012	15	26	62.0 (41.0–75.0)	24	17	18.3 (95% CI, 9.7–21.9)	41.9 (95% CI, 35.1–Nr)	
	39				SOC		13	26	61.0 (39.0–75.0)	17	20	15.3 (95% CI, 11.3–17.4)	30.7 (95% CI, 23.2–40.5)	
Lisheng Xu	90	II	Multicenter	China	GCP	2014–2016	33	57	58.6 ± 9.9	51	38	16.0 (95% CI, 13.9–18.1)	36 (95% CI, 31.7–40.3)	
	89				SOC		23	66	61.0 ± 9.5	52	37	10 (95% CI, 8.7–11.3)	34 (95% CI, 29.1–38.9)	
Chaolun An	45	II	Multicenter	China	GP	2010–2013	25	20	65.72 ± 13.02	17	28	18.0 (95% CI, 15.7–16.2)		34 (95% CI, 28.7.7–39.2)
	45				SOC		25	20	66.89 ± 12.46	16	29	14.0 (95% CI, 11.8–16.2)		32 (95% CI, 26.7–37.2)
Yukio Hosomi,	169	III	Multicenter	Global	GCP	2011–2015	56	114	64.8 ± 7.8	93	69	20.93 (95% CI, 17.94–24.20)		50.90 (95% CI, 41.77–62.50)
	172				SOC		64	108	64.0 ± 8.4 (37–75)	95	67	11.17 (95% CI, 8.97–13.40)		38.80 (95% CI, 31.10–47.33)
S.S. Ramalingam	279	III	Multicenter	Global	O	2014–2016	101	178	64.0 (26.0–85.0)	175	104	18.9 (95% CI, 15.2–21.4)		38.6 (95% CI, 34.5–41.8)
	277				SOC		105	172	64.0 (35.0–93.0)	174	103	10.2 (95% CI, 9.6–11.1)		31.8 (95% CI, 26.6–36.0)
James Chih-Hsin Yang,	129	II	Multicenter	East Asian	GP	2012–2013	64	82	62.0 (33.2–83.6)	65	52	16.2 (95% CI, 12.6–18.7)		43.4 (95% CI, 33.4–50.8)
	66				SOC		24	41	62.0 (40.5–79.5)	40	23	11.1 (95% CI, 9.7–13.8)		36.8 (95% CI, 26.7–42.6).
Kazuhiko Nakagawa	224	III	Multicenter	Global	ER	2016–2018	83	141	65.0 (57.0–71.0)	123	99	19.4 (95% CI, 15.4–21.6)		NR
	225				SOC		83	142	64.0 (56.0–70.0)	120	105	12.4 (95% CI, 11.0–13.5)		NR
N. Yamamoto	75	II	Multicenter	Japan	EB	2011–2012	30	45	67.0 (59.0–73.0)	40	35	16.0 (95% CI, 13.9–18.1)		47
	77				SOC		26	51	67.0 (60.0–73.0)	40	37	9.7 (95% CI, 5.7–11.1)		47.4
Hongyun Zhao	157	III	Multicenter	China	GA	2017–2018	66	91	57.0 (51.0–65.0)	81	74	13.7 (95% CI, 11.9–14.1)		NR
	156				SOC		62	94	60.0 (51.0–65.0)	83	73	10.2 (95% CI, 10.1–11.9)		NR
Qing Zhou	157	III	Multicenter	China	EB	2016–2017	41	71	67.0 (61.0–73.0)	82	75	17.9 (95% CI, 15.2–19.9)		36.2 (95% CI, 32.5–42.4)
	154				SOC		39	73	68.0 (62.0–73.0)	79	75	11.2 (95% CI, 9.7–13.8)		31.6 (95% CI, 27.2–40.0)
Yosuke Kawashima	112	III	Multicenter	Japan	EB	2015– 2016	41	71	67.0 (61.0–73.0)	56	56	16.9 (95% CI, 14.2–21.0)		50.7 (95% CI, 37.3–NE)
	114				SOC		39	73	68.0 (62.0–73.0)	55	57	13.3 (95% CI, 11.1–15.3)		46.2 (95% CI, 38.2–NE)
Maria Carmela Piccirillo	80	III	Multicenter	Italy	EB	2016– 2019	28	52	65.9 (57.9–71.8)	44	32	15.4 (95% CI, 12.2–18.6)		33.3 (95% CI, 24.3–45.1)
	80				SOC		30	50	67.7 (60.7–73.6)	44	34	9.6 (95% CI, 8.2–10.6)		22.8 (95% CI, 18.3–33.0)
Yuqing Lou,Baohui Han	40	II	Monocenter	China	GCP	2011–2015	15	25	< 65.0 (57.5%)	21	19	17.5 (95% CI, 15.3–19.7)		37.9 (95% CI, 17.3–58.6)
	41				SOC		18	23	< 65.0 (43.9%)	21	20	11.9 (95% CI, 9.1–14.6)		25.8 (95% CI, 19.2–32.3)
Vanita Noronha,	174	III	Monocenter	India	GCP	2016–2018	88	96	54.0 (27.0–75.0)	107	109	16.0 (95% CI, 13.5–18.5)		NR
	176				SOC		93	83	56.0 (27.0–78.0)	60	60	8.0 (95% CI, 7.0–9.0)		17 (95% CI, 13.5–20.5)
S. Sugawara	41	II	Multicenter	Japan	GCP	2010–2012	15	26	62.0 (41.0–75.0)	24	17	18.3 (95% CI, 9.7–21.9)		41.9 (95% CI, 35.1–Nr)
	39				SOC		13	26	61.0 (39.0–75.0)	17	20	15.3 (95% CI, 11.3–17.4)		30.7 (95% CI, 23.2–40.)5
Lisheng Xu	90	II	Multicenter	China	GCP	2014–2016	33	57	58.6 ± 9.9	51	38	16.0 (95% CI, 13.9–18.1)		36 (95% CI, 31.7–40.3)
	89				SOC		23	66	61.0 ± 9.5	52	37	10 (95% CI, 8.7–11.3)		34 (95% CI, 29.1–38.9)

EB = Erlotinib+Bevacizumab, ER = Erlotinib+Ramucirumab, GA = Gefitinib+Apatinib, GCP = SOC+Carboplatin+Pemetrexed, GP = Gefitinib+Pemetrexed, O = Osimertinib, OS = overall survival, PFS = progression-free survival, SOC = Gefitinib/Erlotinib/Icotinib, TKIs = tyrosine kinase inhibitor.

### 3.4. Network meta-analysis

**Figure 3.**
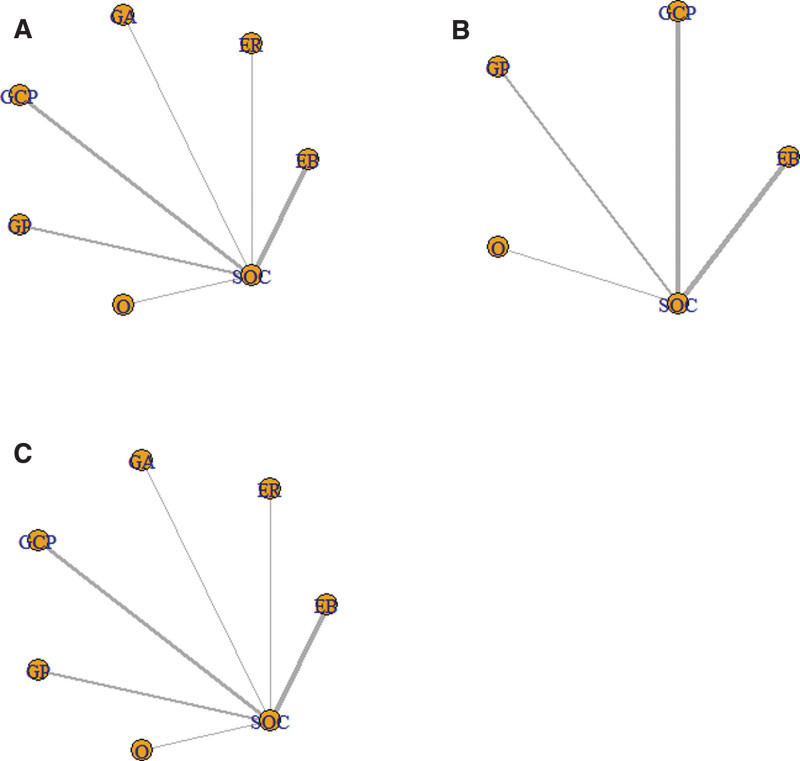
A. PFS of network plot. B. OS of network plot. C. TEAEs of network plot. EB = Erlotinib+Bevacizumab, ER = Erlotinib+Ramucirumab, GA = Gefitinib+Apatinib, GCP = SOC+Carboplatin+Pemetrexed, GP = Gefitinib+Pemetrexed, O = Osimertinib, OS = overall survival, PFS = progression-free survival, SOC = Gefitinib/Erlotinib/Icotinib, TEAEs = treatment emergent adverse events, TKIs = tyrosine kinase inhibitor.

The network diagram established by the network meta-analysis (NMA) is shown in Figure 3A–C. The NMA results are presented in Table 2. The PFS of osimertinib, GP, ER, EB, and GCP was better than that of SOC, the results were statistically different. In terms of PFS, osimertinib ranked first in the league table, but the difference between osimertinib and the other 4 combination regimens was not statistically significant. In terms of OS, GCP was more effective and statistically significant than SOC was. The GCP ranked first in the league table, and there were no significant differences between the EB, GP, GCP, and osimertinib groups. In terms of grade 3 and above TEAEs, the risk of TEAEs caused by SOC and osimertinib was significantly lower than that of GCP, EB, and GA-based regimens, and the difference was statistically significant. There were no significant difference in TEAEs between GCP and ER or GP. The results of all the comparisons are shown in Figure 3A–C. Not all outcomes were provided in any of the studies.

**Table 2:** Results of network meta-analysis.

A. Hazard ratios (HR) with 95% confidence interval (CI) for progression-free survival (PFS)								
SOC								
−0.33 (−0.67, 0.01)	GA							
**−0.39 (−0.71, −0.08**)	−0.07 (−0.54, 0.41)	GP						
**−0.53 (−0.86, −0.2**)	−0.2 (−0.67, 0.27)	−0.14 (−0.59, 0.31)		ER				
**−0.54 (−0.73, −0.34**)	−0.21 (−0.61, 0.19)	−0.15 (−0.51, 0.23)		−0.01 (−0.38, 0.37)	EB			
**−0.64 (−0.81, −0.46**)	−0.31 (−0.69, 0.08)	−0.25 (−0.6, 0.13)		−0.11 (−0.47, 0.27)	−0.1 (−0.36, 0.17)		GCP	
**−0.78 (−1.08, −0.48**)	**−0.45 (−0.9, 0**)	−0.39 (−0.82, 0.05)		−0.25 (−0.69, 0.19)	−0.24 (−0.6, 0.12)		−0.14 (−0.49, 0.2)	O
B. Hazard ratios (HR) with 95% confidence interval (CI) for overall survival (OS)								
SOC								
−0.14 (−0.37, 0.09)	EB							
−0.19 (−0.6, 0.24)	−0.05 (−0.52, 0.44)	GP						
−0.22 (−0.58, 0.14)	−0.08 (−0.51, 0.35)	−0.03 (−0.59, 0.51)		O				
**−0.38 (−0.64, −0.14**)	−0.25 (−0.6, 0.1)	−0.19 (−0.69, 0.29)		−0.16 (−0.61, 0.27)	GCP			
C. Odds ratios (OR) with 95% confidence interval (CI) for treatment−emergent adverse eventss (TEAEs)								
O								
−0.36 (−1.42, 0.71)	SOC							
−0.84 (−2.31, 0.67)	−0.47 (−1.51, 0.57)		ER					
−1.06 (−2.49, 0.35)	−0.7 (−1.64, 0.23)		−0.23 (−1.63, 1.17)	GP				
**−1.25 (−2.51, −0.02**)	**−0.89 (−1.57, −0.25**)		−0.41 (−1.67, 0.8)	−0.19 (−1.35, 0.94)	GCP			
**−1.26 (−2.43, −0.04**)	**−0.89 (−1.43, −0.34**)		−0.42 (−1.59, 0.77)	−0.19 (−1.27, 0.91)	−0.01 (−0.83, 0.89)	EB		
**−1.74 (−3.23, −0.22**)	**−1.37 (−2.43, −0.31**)		−0.9 (−2.39, 0.6)	−0.67 (−2.09, 0.75)	−0.49 (−1.71, 0.8)	−0.48 (−1.67, 0.71)		GA

For survival outcomes (OS, PFS).

An HR below 0 favors the column-defining treatment. For safety (TEAEs) above 0 favors the column-defining treatment. Comparisons with differences of statistical significance (*P* < .05) are highlighted in bold format.

EB = Erlotinib+Bevacizumab, ER = Erlotinib+Ramucirumab, GA = Gefitinib+Apatinib, GCP = SOC+Carboplatin+Pemetrexed, GP = Gefitinib+Pemetrexed, O = Osimertinib, OS = overall survival, PFS = progression-free survival, SOC = Gefitinib/Erlotinib/Icotinib, TKIs = tyrosine kinase inhibitor.

### 3.5. Rank probabilities

**Figure 4.**
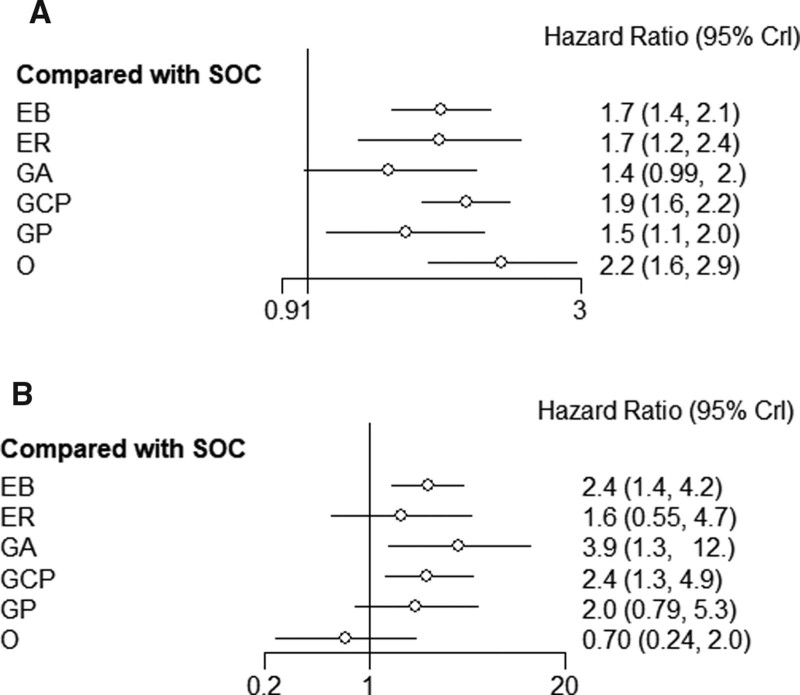
A. PFS results for each treatment measure relative to SOC. B. OS results for each treatment measure relative to SOC. C. TEAEs results for each treatment measure relative to SOC. OS = overall survival, PFS = progression-free survival, SOC = Gefitinib/Erlotinib/Icotinib, TEAEs = treatment emergent adverse events.

Table 3 and Figures S1–S3, Supplemental Digital Content, http://links.lww.com/MD/J361, which is the Rank Probabilities graph, including PFS, OS, TEAEs. shows the ranking indicating the probability of being the best treatment, the second best, third best, and so on, among all the treatment regimens. Agents with greater histogram values were associated with greater probabilities of better outcomes. Based on Figure 4A–C, the cumulative probabilities of being the most efficacious treatments were (PFS, OS, TEAEs): O (73%, 16%, 0%, 0%), GCP (14%, 64%, 10%, 16%), GP (2%, 17%, 8%), and EB (3%, 3%, 8%); ER (5%, NA, 4%); GA (1%, NA, 69%). However, the outcomes of ER were not assessable. Osimertinib may be the best treatment option due to its better PFS and tolerability. GCP and GP are also 2 more effective regimens, but the risk of TEAEs is relatively high.

**Table 3:** Rank probabilities.

Progression-free survival (PFS)								
	V1	V2		V3	V4	V5	V6	V7
EB	0.03	0.13		0.31	0.33	0.15	0.04	0
ER	0.08	0.17		0.23	0.25	0.18	0.08	0
GA	0.01	0.03		0.05	0.11	0.26	0.52	0.03
GCP	0.14	0.46		0.26	0.1	0.03	0	0
GP	0.02	0.05		0.09	0.17	0.36	0.32	0.01
O	**0.73**	0.16		0.06	0.03	0.01	0	0
SOC	0	0		0	0	0	0.04	**0.96**
**Overall survival (OS**)								
	**V1**	**V2**	**V3**		**V4**	**V5**		
EB	0.03	0.17	0.36		0.35	0.09		
GCP	**0.64**	0.26	0.08		0.02	0		
GP	0.17	0.23	0.24		0.19	0.18		
O	0.16	0.34	0.28		0.15	0.08		
SOC	0	0	0.05		0.29	**0.65**		
**Treatment-emergent adverse events (TEAEs**)								
	**V1**	**V2**		**V3**	**V4**	**V5**	**V6**	**V7**
EB	0.08	0.31		0.33	0.21	0.07	0.01	0
ER	0.04	0.08		0.1	0.21	0.4	0.1	0.06
GA	**0.69**	0.14		0.08	0.05	0.03	0.01	0
GCP	0.1	0.29		0.3	0.21	0.08	0.01	0
GP	0.08	0.17		0.17	0.28	0.22	0.05	0.02
O	0	0.01		0.01	0.02	0.06	0.13	**0.76**
SOC	0	0		0	0.01	0.14	0.69	0.15

Best and worst are bold.

EB = Erlotinib+Bevacizumab, ER = Erlotinib+Ramucirumab, GA = Gefitinib+Apatinib, GCP = SOC+Carboplatin+Pemetrexed, GP = Gefitinib+Pemetrexed, O = Osimertinib, SOC = Gefitinib/Erlotinib/Icotinib, TKIs = tyrosine kinase inhibitor.

### 3.6. Inconsistency assessment

There are no independent closed loops in the network plot of the NMA. No consistency checks are required.

## 4. Discussion

This new network meta-analysis evaluated the efficacy and tolerability of all first-line therapies of TKI-based combination regimens (EGFR-TKI combination therapy) versus osimertinib in advanced EGFR-mutated NSCLC, which is currently the mainstream recognized low toxicity, effective preferred protocol, based on the FLAURA study.^[18,19]^ The results showed that osimertinib provided better PFS and lower TEAEs compared to SOC. Compared with SOC regimen, GCP regimen had better OS improvement, and the results were statistically significant. According to the NMA results, osimertinib seems to be the preferred first-line treatment for patients with poor performance status or advanced age, but for patients with good performance score and tolerance to chemotherapy combination, GCP^[12,32,33]^ regimen may have better potential.ER^[25]^ is another more effective treatment for NMA. Order according to treatment. ER regimen also had the lowest TEAEs among the combination regimens.ER^[25]^ regimen also has superior median PFS, but the mOS is not mature; therefore, the final results can be expected. Additional, the EB regimen is currently considered to prolong PFS, especially in patients with exon 21 L858R, but does not prolong survival, which may be related to the lack of direct antitumor therapy.^[34]^[Bibr R15]

This network meta-analysis has some limitations. First, the current OS data for ER regimens are not mature, and can not be compared. In addition, subsequent multiple lines of therapy may not further assess the OS outcomes. Second, the use of first-generation EGFR-TKIs is not considered to be different in efficacy. However, in combination therapy, similar drugs may not be able to replace one another. Third, except for a few combination regimens and osimertinib, which were double-blind, most combination therapies were open-label, which may have introduced some bias. Fourth, the established networks lack sufficient direct comparisons between TKIs. Fifth, data were not available for sufficient subgroup populations to allow for additional subgroup analyses.

## 5. Conclusions

Osimertinib has the lowest side effects and provides better PFS first-line treatment in advanced EGFR-mutated NSCLC.GCP is the best regimen for OS, but its toxicity limits its application, and it may be the first choice for patients with higher survival requirements.

## Acknowledgments

The authors thank Research staff for her technical assistance for this study.

## Author contributions

**Conceptualization:** Yang Lei.

**Formal analysis:** Qing Li.

**Investigation:** Jia Duan.

**Methodology:** Jia Duan, Qiong Zhang, Qing Li.

**Project administration:** Qiong Zhang.

**Resources:** Qiong Zhang.

**Software:** Yang Lei.

**Writing – original draft:** Yang Lei.

**Writing – review & editing:** Yang Lei.

## Supplementary Material




