# Supplementary material for: Comparation of EGFR-TKI (EGFR tyrosine kinase inhibitors) combination therapy and osimertinib for untreated EGFR-mutated advanced non-small cell lung cancers: A systematic review and network meta-analysis
Source: Medicine (Baltimore). 2023 Jul 28;102(30):e34483. doi: 10.1097/MD.0000000000034483 (PMC10378737; doi:10.1097/MD.0000000000034483)
Supplement: Supplementary file 1 [file medi-102-e34483-s001.pdf]

Table S1.Characteristics and rerults

Study	Treatment	ORR(ORR%)	PFS/HR95CI	OS/HR 95CI	Adverse Events
Chaolun An	Gefitinib+Pemetrexed	36(80.0%)	0.70（0.36-1.35）	0.97(0.50-1.88)	10(22.2%)
	Gefitinib	33（73.3%）			9(20.0%)
Yukio Hosomi,	gefitinib+Carboplatin+Pemetrexed	142(84.0%)	0.49(0.39-0.62)	0.72 (0.55-0.95)	111(65.3%)
	Gefitinib	115(67.0%)			53(31.0%)
S.S. Ramalingam	Osimertinib	223(80.0%)	0.46(0.37–0.57)	0.80(0.64–1.00)	95(34.0%)
	Gefitinib/Erlotinib	211(76.0%)			125(45.0%)
James Chih-Hsin Yang,	Gefitinib+Pemetrexed	101(80%)	0.67(0.50-0.90)	0.77(0.50-1.20)	32(25.4%)
	Gefitinib	48(74%)			6(9.2%)
Kazuhiko Nakagawa	Erlotinib+Ramucirumab	171(76%)	0.59(0.46–0.76]	NR	159(72.0%)
	Erlotinib	168(75%)			121(54.0%)
N. Yamamoto	Erlotinib+Bevacizumab	52(69.0%)	0.54(0.36–0.79)	0.81（0.53–1.23)	68 (91.0%)
Takashi Seto	Erlotinib	49(63.0%)			41 (53.0%)
Hongyun Zhao	Gefitinib+Apatinib	121(77.1%)	0.71(0.54–0.95)	NR	132(84.1%)
	Gefitinib	115(73.7%)			58 (37.7%)
Qing Zhou	Erlotinib+Bevacizumab	132(86.8%)	0.55(0.41–0.73)	0.92(0.69–1.23)	86 (54.8%)
	Erlotinib	127(84.7%)			40 (26.1%)
Yosuke Kawashima	Erlotinib+Bevacizumab	81(72.0%)	0,61(0.42–0.88)	1.01(0.68–1.49)	98 (88%)
	Erlotinib	74(66.0%)			53 (46%)
Maria Carmela Piccirillo	Erlotinib+Bevacizumab	56(70.0%)	0.66(0.47–0.92）	0.72(0.47–1.10)	45(56%)
	Erlotinib	40(50.0%)			39(49%)
Yuqing Lou,Baohui Han	Gefitinib+Pemetrexed+Carboplatin	33（82.5%）	0.48(0.29–0.78)	0.56(0.34–0.91)	12（30%）
	Gefitinib	27（65.9%）			5（12.2%）

Vanita Noronha,	Gefitinib+Carboplatin+Pemetrexed	131（75.3%）	0.51(0.39-0.66)	NR	123(75%)
	Gefifitinib	110（62.5%）			84(49.4%)
S. Sugawara	Gefitinib+Carboplatin+Pemetrexed	36(87.8%)	0.71(0.42-1.20)	0.51(0.26-0.99]	NR
	Gefitinib	33(84.6%)			NR
Lisheng Xu	Icotinib+Pemetrexed+Carboplatin	70(77.8%)	0.59(0.42–0.84)	0.81(0.54–1.22)	NR
	Icotinib	57(64.0%)			NR
