# Supplementary material for: Comparation of EGFR-TKI (EGFR tyrosine kinase inhibitors) combination therapy and osimertinib for untreated EGFR-mutated advanced non-small cell lung cancers: A systematic review and network meta-analysis
Source: Medicine (Baltimore). 2023 Jul 28;102(30):e34483. doi: 10.1097/MD.0000000000034483 (PMC10378737; doi:10.1097/MD.0000000000034483)

Figure S1.Rank Probabilities of PFS

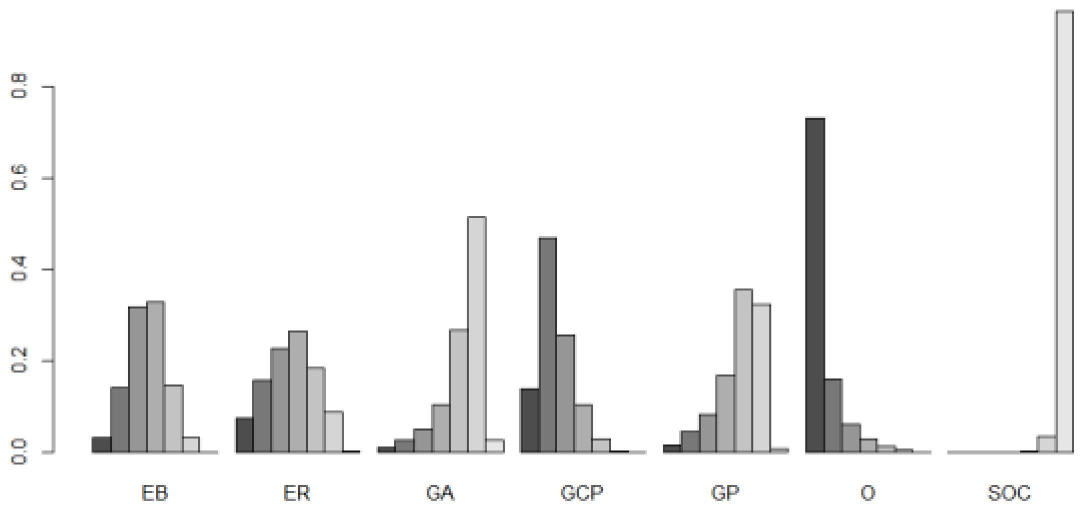

Figure S2. Rank Probabilities of OS

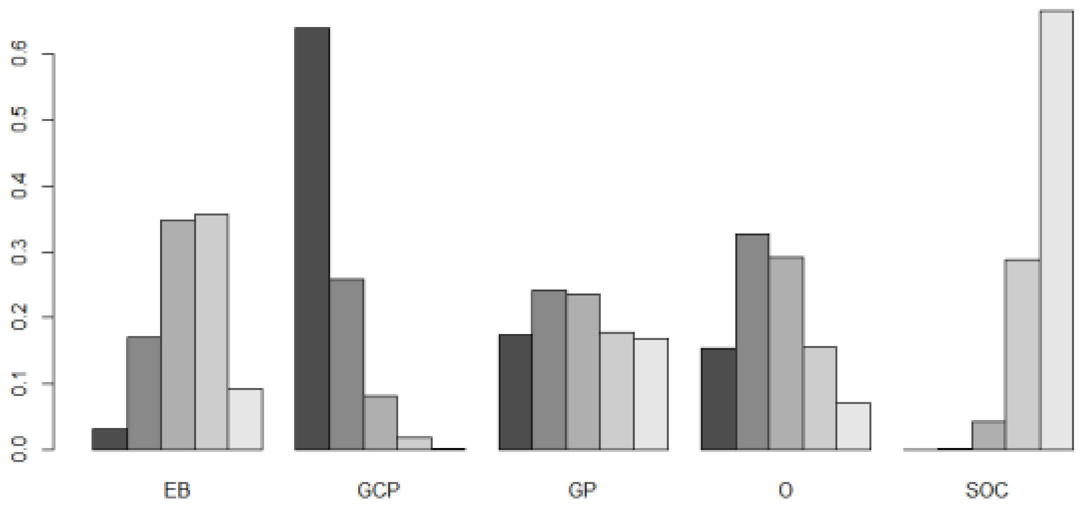

Figure S3. Rank Probabilities Of TEAEs

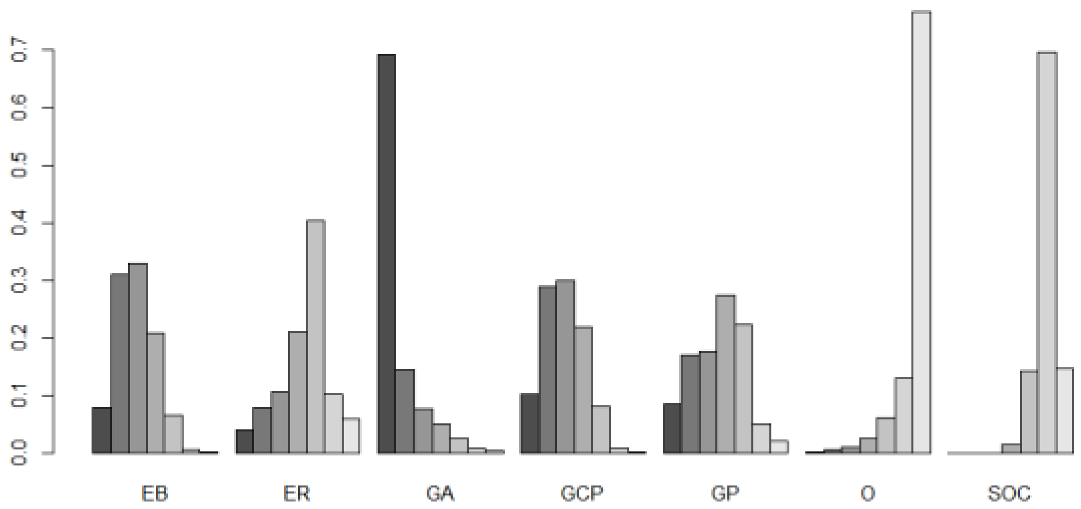

Supplement: Supplementary file 2 [file medi-102-e34483-s002.pdf]
